# Optimum timing of lung resection surgery following SARS‐CoV‐2 infection for non‐small cell lung cancer

**DOI:** 10.1002/cam4.6891

**Published:** 2024-01-02

**Authors:** Yanbo Yang, Lingli Niu, Yunke Zhu, Zhu Wu, Liang Xia, Congjia Xiao, Xu Shen, Xin Xiao, Conglin Tian, Feng Lin

**Affiliations:** ^1^ Department of Thoracic Surgery, West China Hospital Sichuan University Chengdu China; ^2^ Department of Thoracic Surgery, Shangjin Branch of Western China Hospital Sichuan University Chengdu China; ^3^ Western China Collaborative Innovation Center for Early Diagnosis and Multidisciplinary Therapy of Lung Cancer Sichuan University Chengdu China

**Keywords:** coronavirus disease 2019 (COVID‐19), non‐small cell lung cancer (NSCLC), optimum timing, severe acute respiratory syndrome coronavirus 2 (SARS‐CoV‐2), surgical outcome

## Abstract

**Background:**

The impact of severe acute respiratory syndrome coronavirus 2 (SARS‐CoV‐2) infection on postoperative recovery of non‐small cell lung cancer (NSCLC) is need to be understood, thereby informing the optimal timing of surgical decision‐making during the COVID‐19 pandemic for NSCLC patients. This study reports the postoperative outcomes of surgical NSCLC patients with preoperative SARS‐CoV‐2 infection.

**Method:**

This single‐center retrospective cohort study included 241 NSCLC patients who underwent lobectomy or sub‐lobectomy between December 1, 2022 and February 14, 2023. Surgical outcomes of patients with preoperative SARS‐CoV‐2 infection (stratified by the time from diagnosis of SARS‐CoV‐2 infection to surgery) were compared with those without preoperative SARS‐CoV‐2 infection. The primary outcomes were total postoperative complications and postoperative pulmonary complications (PPCs), the secondary outcomes included operation time, total postoperative drainage and time, length of hospital stay (LOS), 30‐day and 90‐day postoperative symptoms.

**Results:**

This study included 153 (63.5%) patients with preoperative SARS‐CoV‐2 infection and 88 (36.5%) patients without previous SARS‐CoV‐2 infection. In patients with a preoperative SARS‐CoV‐2 diagnosis, the incidence of total postoperative complications (OR, 3.00; 95% CI, 1.12–8.01; *p* = 0.028) and PPCs (OR, 4.20; 95% CI, 1.11–15.91; *p* = 0.035) both increased in patients infected having surgery within 2 weeks compared with non‐infection before surgery. However, patients who underwent lung resection more than 2 weeks after SARS‐CoV‐2 diagnosis had a similar risk of postoperative complications and surgical outcomes with those non‐infection before surgery.

**Conclusion:**

This is the first study to provide evidence regarding the optimum timing of lung resection surgery and assessing early outcomes after surgery in NSCLC patients with SARS‐CoV‐2 infection. Our study documents that the SARS‐CoV‐2 infection did not complicate surgical procedures for lung cancer, and suggest that lung surgery should be postponed at least 2 weeks after SARS‐CoV‐2 infection for NSCLC patients.

## 1 INTRODUCTION

Patients with perioperative severe acute respiratory syndrome coronavirus 2 (SARS‐CoV‐2) infection are at increased risk of death and pulmonary complications following surgery,^1^, ^2^, ^3^, ^4^, ^5^ probably due to the release of pro‐inflammatory cytokine, immunosuppressive responses to surgery and increased use of mechanical ventilation.^6^, ^7^ Patients with lung cancer represent a particularly vulnerable population during this period, and relevant information has emerged regarding a higher risk of poor outcomes with pulmonary complications and mortality in patients.^8^, ^9^, ^10^ With the SARS‐CoV‐2 pandemic continues to rage, surgical resection would become more common for lung cancer patients who previously had SARS‐CoV‐2 infection. To avoid a deferred public health crisis of unnecessary cancer‐related deaths, lung cancer resections as lifesaving surgery should not be delayed for too long time and should be triaged based on the clinical features of the tumor and allowing prioritization.^10^, ^11^, ^12^, ^13^ As such, clinical guidelines suggest postponing non‐emergency surgery for patients with preoperative SARS‐CoV‐2 infection, but the recommended times vary^14^, ^15^, ^16^, ^17^, ^18^ and most of these were based solely on expert opinion or limited clinical data.

The international cohort studies recommend that, whenever possible, surgery should be postponed in patients with SARS‐CoV‐2 infection to mitigate the risk of postoperative pulmonary complications and mortality.^1^, ^2^, ^5^ However, few evidence regarding the optimum timing of lung resection surgery following SARS‐CoV‐2 infection for lung cancer patients. Thus, more real‐world granular data were needed urgently in order to enable surgeons to make evidence‐based decisions for patients during the pandemic. The aim of this study was to determine the optimal timing of lung resection surgery following SARS‐CoV‐2 infection for NSCLC patients.

## 2 MATERIALS

### 2.1 Study design and patients

This study retrospectively included patients who diagnosed with NSCLC and underwent elective lobectomy or sub‐lobectomy between December 1, 2022 and February 14, 2023, at Shangjin Branch of Western China Hospital, Sichuan University. Emergency surgery and non‐pulmonary surgery patients were excluded and benign tumor, pulmonary bulla, and pneumothorax were also excluded as the aim of this study was focused on lung cancer patients.

**FIGURE 1:**
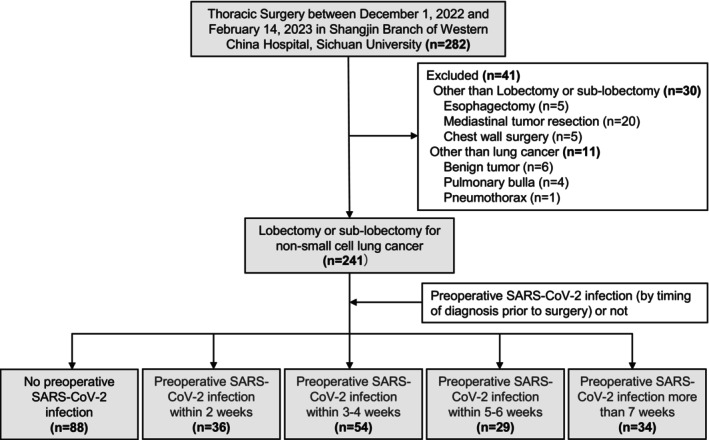
Patient inclusion flow chart.

Patients were classified as having preoperative SARS‐CoV‐2 infection or non‐infection based on positive RT‐PCR nasopharyngeal swab taken before surgery, the former takes the sampling time of positive SARS‐CoV‐2 nucleic acid test as the date of diagnosis and was divided into the following groups by timing of diagnosis prior to surgery: within 2 weeks, 3–4 weeks, 5–6 weeks, and more than 7 weeks (Figure 1).

This study was approved by the Institutional Review Board (IRB) of West China Hospital, Sichuan University. Given its retrospective nature, informed consent was waived. We collected only routine, anonymized data with no change to clinical care pathways. This study was compliant with guidelines for the reporting of observational studies.^19^

### 2.2 Data collection and procedures

Time from the diagnosis of SARS‐CoV‐2 infection to the day of surgery was collected as a categorical factor and pre‐determined to be analyzed in the following categories: within 2 weeks, 3–4 weeks, 5–6 weeks and more than 7 weeks. Laboratory testing for SARS‐CoV‐2 infection was based on viral RNA detection by quantitative RT‐PCR; data were captured in all patients whether or not having SARS‐CoV‐2 relative symptoms, both non‐respiratory and respiratory symptoms, and patients were classified as follows: symptomatic and asymptomatic. Baseline and demographic variables were captured from electronic medical record system, including age, gender (male, female), smoking history (nonsmoker, current or former smoker), body mass index (BMI), and Charlson comorbidity index (CCI),^20^ American Society of Anesthesiologists (ASA) physical status classification (ASA at the time of surgery was classified as Grades 1–2 and Grades 3–5), the timing of SARS‐CoV‐2 diagnosis (either preoperative or postoperative), surgical modalities (lobectomy or sub‐lobectomy), surgical side (right or left), histologic types (adenocarcinoma or non‐adenocarcinoma), pathologic stage (the 8th edition of American Joint Committee on Cancer lung cancer staging system, including pT, pN, and pTNM status) (Table 1).

**TABLE 1:** Baseline characteristics for patients undergoing lung cancer surgery stratified by time from diagnosis of SARS‐CoV‐2 infection. Values are number (proportion).

Patient characteristics no. (%)	No preoperative SARS‐CoV‐2 infection, (*n* = 88)	Preoperative SARS‐CoV‐2 infection (by timing of diagnosis prior to surgery)				*p*‐value
		0–2 weeks, (*n* = 36)	3–4 weeks, (*n* = 54)	5–6 weeks, (*n* = 29)	≥7 weeks, (*n* = 34)	
Age at surgery						0.415^a^
Age < 60	54 (61.4)	25 (69.4)	41 (75.9)	19 (65.5)	25 (73.5)	
Age ≥ 60	34 (38.6)	11 (30.6)	13 (24.1)	10 (34.5)	9 (26.5)	
Gender						0.710^a^
Female	57 (64.8)	22 (61.1)	35 (64.8)	19 (65.5)	26 (75.5)	
Male	31 (35.2)	14 (38.9)	19 (35.2)	10 (34.5)	8 (23.5)	
BMI						0.711^b^
<18.5	1 (1.1)	1 (2.8)	2 (3.7)	2 (6.9)	3 (8.8)	
18.5–25	66 (75.0)	27 (75.0)	39 (72.2)	21 (72.4)	24 (70.6)	
>25	21 (23.9)	8 (22.2)	13 (24.1)	6 (20.7)	7 (20.6)	
CCI						0.027^b^
0	62 (72.7)	24 (66.7)	49 (90.7)	20 (69.0)	30 (88.2)	
1	21 (23.9)	10 (27.8)	3 (5.6)	8 (27.6)	3 (8.8)	
≥2	3 (3.4)	2 (5.6)	2 (3.7)	1 (3.4)	1 (2.9)	
ASA physical status						0.731^b^
1–2	83 (94.3)	33 (91.7)	52 (96.3)	26 (89.7)	32 (94.1)	
3–5	5 (5.7)	3 (8.3)	2 (3.7)	3 (10.3)	2 (5.9)	
COVID‐19 syndrome						0.889^b^ ^,^ *
Symptomatic	–	32 (88.9)	50 (92.6)	27 (93.1)	32 (94.1)	
Asymptomatic	–	4 (11.1)	4 (7.4)	2 (6.9)	2 (5.9)	
Smoking history						0.252^a^
Nonsmoker	62 (70.5)	29 (80.6)	42 (75.9)	20 (69.0)	30 (88.2)	
Current or former smokers	26 (29.5)	7 (19.4)	13 (24.1)	9 (31.0)	4 (11.8)	
Operation side						0.450^a^
Left side	34 (38.6)	9 (25.0)	23 (42.6)	9 (31.0)	11 (36.8)	
Right side	54 (61.4)	27 (75.0)	31 (57.4)	20 (69.0)	23 (63.2)	
Surgical modalities						0.545^a^
Lobectomy	55 (62.5)	19 (52.8)	36 (66.7)	15 (51.7)	22 (64.7)	
Sub‐lobectomy	33 (37.5)	17 (47.2)	18 (33.3)	14 (48.3)	12 (35.3)	
Histologic types						0.663^a^
Adenocarcinoma	75 (85.2)	29 (80.6)	43 (79.6)	24 (82.8)	25 (73.5)	
Non‐adenocarcinoma	13 (14.8)	7 (19.4)	11 (20.4)	5 (17.2)	9 (26.5)	
pT stage						0.526^b^
T_1_	77 (87.5)	32 (88.9)	49 (90.7)	27 (93.1)	27 (79.4)	
T_2_–T_4_	11 (12.5)	4 (11.1)	5 (9.3)	2 (6.9)	7 (20.6)	
pN stage						0.910^b^
N_0_	87 (98.9)	35 (97.2)	53 (98.1)	29 (100.0)	34 (100.0)	
N_1_–N_2_	1 (1.1)	1 (2.8)	1 (1.9)	0 (0.0)	0 (0.0)	
pTNM status						0.796^b^
Stage I	81 (92.0)	35 (97.2)	52 (96.3)	28 (96.6)	33 (97.1)	
Stage II–III	7 (8.0)	1 (2.8)	2 (3.7)	1 (3.4)	1 (2.9)	

Abbreviations: ASA, American Society of Anesthesiologists; BMI, body mass index; CCI, Charlson Comorbidity Index; COVID‐19, coronavirus disease 2019; LOS, length of hospital stay; PPCs, postoperative pulmonary complications; SARS‐CoV‐2, the severe acute respiratory syndrome coronavirus 2.

^a^ Pearson chi‐square.

^b^ Fisher's except test.

* Patients without preoperative SARS‐CoV‐2 infection did not include in the analysis.

The surgical outcomes and postoperative complications were also collected for each patient, including surgery duration, total postoperative complications and postoperative pulmonary complications (PPCs), total length of hospital stay (LOS), postoperative LOS, total postoperative drainage, duration of thoracic drainage, prolonged chest drainage or air leakage ≥5 days, operative mortality, convert to open surgery, and amount of blood transfusion. Patients were followed‐up either in person or by telephone at Day 30 and Day 90, and symptoms of patients were recorded (Table 2).

**TABLE 2:** Surgical outcomes and postoperative data for patients undergoing lung cancer surgery stratified by time from diagnosis of SARS‐CoV‐2 infection.

Patient characteristics mean ± SD or no. (%)	No preoperative SARS‐CoV‐2 infection, (*n* = 88)	Preoperative SARS‐CoV‐2 infection (by timing of diagnosis prior to surgery)			
		0–2 weeks, (*n* = 36)	3–4 weeks, (*n* = 54)	5–6 weeks, (*n* = 29)	≥7 weeks, (*n* = 34)
Surgery duration (min)	73.3 ± 33.4	78.2 ± 32.2	74.4 ± 33.0	67.8 ± 25.8	62.9 ± 23.5
Convert to open surgery	0 (0.0)	0 (0.0)	0 (0.0)	0 (0.0)	0 (0.0)
Blood transfusion	0 (0.0)	0 (0.0)	0 (0.0)	0 (0.0)	0 (0.0)
Operative mortality	0 (0.0)	0 (0.0)	0 (0.0)	0 (0.0)	0 (0.0)
Postoperative complications					
Total complications	10 (11.4)	10 (27.8)	9 (16.7)	6 (20.7)	2 (5.9)
Complication Clavien–Dindo ≥grade 2	0 (0)	1 (2.8)	2 (3.7)	1 (3.4)	0 (0)
PPCs	4 (4.5)	6 (16.7)	5 (9.3)	2 (6.9)	1 (2.9)
Total postoperative drainage (mL)	497.4 ± 351.1	796.81 ± 1239.6	665.2 ± 931.2	604.3 ± 784.5	440.7 ± 405.4
Duration of thoracic drainage (days)	3.0 ± 1.3	4.1 ± 3.8	3.6 ± 2.6	3.3 ± 2.8	2.7 ± 1.2
PCD or air leakage ≥5 days	8 (9.1)	7 (19.4)	11 (20.4)	5 (17.2)	2 (5.9)
LOS (days)					
Total LOS	9.6 ± 2.6	11.2 ± 5.7	9.3 ± 2.9	8.8 ± 3.3	8.2 ± 1.7
Postoperative LOS	4.9 ± 2.0	6.1 ± 3.9	5.0 ± 2.6	4.4 ± 2.8	4.0 ± 1.3
30‐day postoperative symptoms	39 (44.3)*	20 (55.6)	19 (35.2)	7 (24.1)	3 (8.8)
90‐day postoperative symptoms	8 (9.1)	3 (8.3)	6 (11.1)	2 (6.9)	1 (2.9)

Abbreviations: COVID‐19, coronavirus disease 2019; LOS, length of hospital stay; PCD, prolonged chest drainage; PPCs, postoperative pulmonary complications; SARS‐CoV‐2, the severe acute respiratory syndrome coronavirus 2; SD, standard deviation.

* Eighteen patients without preoperative SARS‐CoV‐2 infection became infected with SARS‐CoV‐2 within 1 month after surgery included in the analysis.

### 2.3 Outcome measures

The primary outcomes measure were total postoperative complications and PPCs. Total postoperative complications included any events of PPCs, bronchopleural fistula (BPF) or/and empyema, pulmonary embolism, chylothorax, and other complications (such as reoperation, postoperative hemorrhage, incisional infection or/and nonunion, subcutaneous emphysema, pleural re‐intubation/postoperative thoracic puncture, arrhythmia, cardiocerebrovascular accident, and gastrointestinal disorders). PPCs included acute respiratory distress syndrome (ARDS), pneumonia and unexpected postoperative ventilation.^1^, ^21^ In addition, even if there were no signs and symptoms of pneumonia, the only occurrence of ground‐glass opacities in the lungs after surgery and confirmed by chest computed tomography (CT) was also classified as PPCs in the study, because these are the most common pulmonary complications related to COVID‐19 in medical patients.^22^, ^23^

The secondary outcomes included operation time, total postoperative drainage, thoracic drainage time, LOS, 30‐day and 90‐day postoperative symptoms. Symptoms were classified into surgery‐related symptoms and COVID‐19 related symptoms, which may includ any of the following: abnormal chest x‐ray or CT scan performed, joint pain, back pain, muscular pain, sore muscles, headache, sleep problems, palpitations, dizziness, anosmia, fatigue, poor attention or concentration, hearing impairment, constipation, skin problems, sensory symptoms (pins and needles, tingling, or burning sensation), stomach pain, chest pain, cough, breathing difficulties, diarrhea, and other symptoms.^24^

### 2.4 Statistical analysis

Categorical variables were described as absolute and relative frequencies, while quantitative variables were described with mean and standard deviation (SD). ANOVA was used to compare quantitative variables of differences between groups. The categorical variables between the two groups were compared using the Pearson chi‐squared test or Fisher's exact test. The independent risk factors for patients were identified by Logistic regression model. Multilevel logistic regression was used to calculate odds ratios (ORs) and 95% CIs. Models included factors that occurred before the outcome of interest. For all comparisons, a *p*‐value of 0.05 was used to define statistical significance (all with double tails). Statistical tests were performed using the IBM SPSS statistical software (IBM Corp. Released 2015. IBM SPSS Statistics for Windows, Version 25.0. Armonk, NY: IBM Corp.).

## 3 RESULTS

A total of 241 patients were included in this study, including 153 (63.5%) had a preoperative SARS‐CoV‐2 diagnosis and 88 (36.5%) without previous SARS‐CoV‐2 infection. At the time of data analysis (May 15, 2023), 90‐day follow‐up has been reached for all patients. Among patients with preoperative SARS‐CoV‐2 infection, the time from SARS‐CoV‐2 diagnosis to surgery was 0–2 weeks in 36 patients (23.5%), 3–4 weeks in 54 patients (35.3%), 5–6 weeks in 29 patients (19.0%), and ≥7 weeks in 34 patients (22.2%). Detailed characteristics and outcomes of the patients are listed in Tables 1 and 2. Of all patients included in this study, there was no death, no conversion to thoracotomy, and no blood transfusion. There is no significant difference in baseline characteristics between patients with preoperative SARS‐CoV‐2 infection and those did not have SARS‐CoV‐2 infection before surgery. We need to explain that 18 patients who did not have SARS‐CoV‐2 infection before surgery but were infected with SARS‐CoV‐2 outside the hospital within 1 month after surgery were not excluded, and all of these patients were asymptomatic at the time of surgery.

**FIGURE 2:**
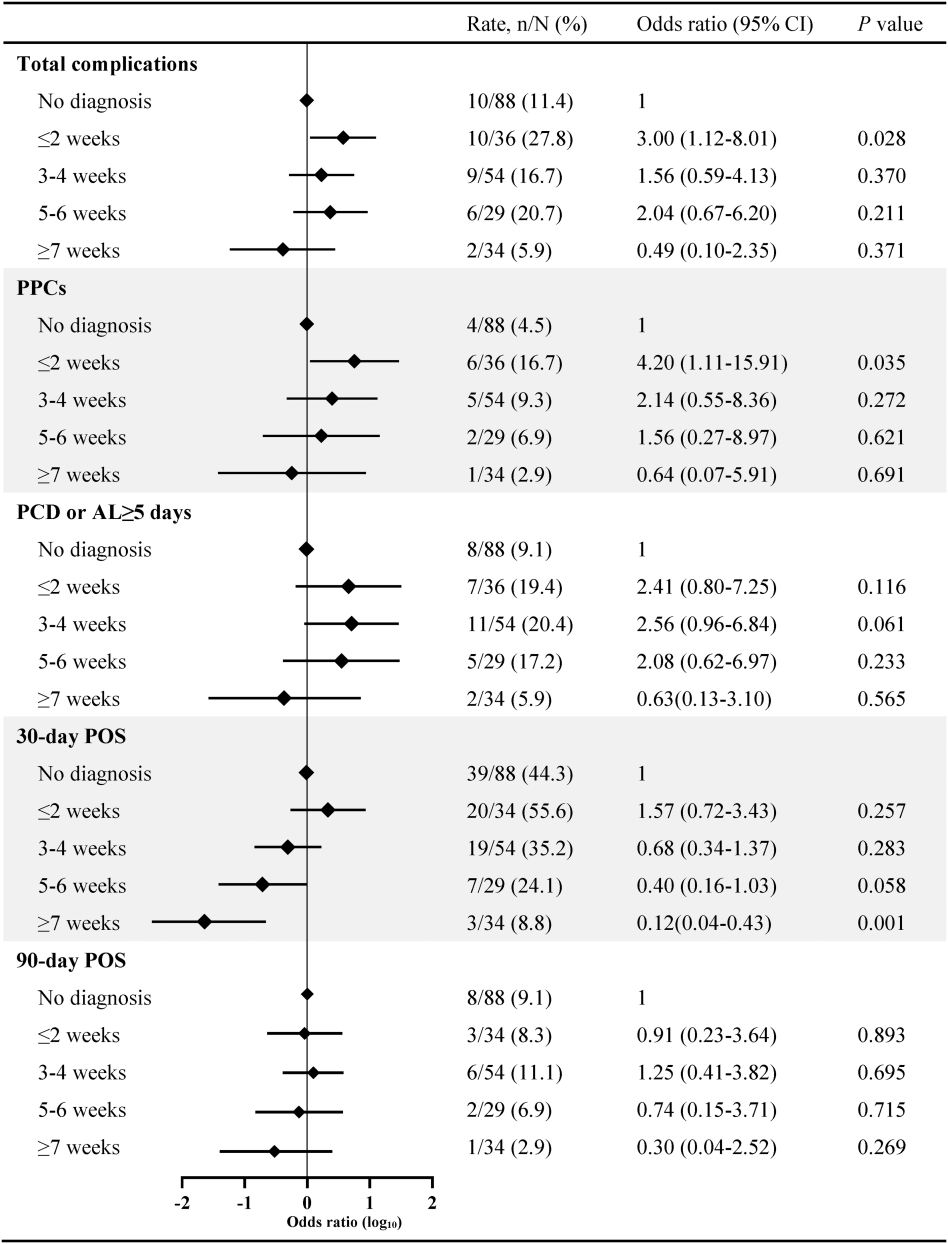
Comparison of postoperative complications between patients with and without preoperative SARS‐CoV‐2 infection diagnosed (preoperative infected patients grouped by timing of diagnosis prior to surgery). AL, air leakage; PCD, prolonged chest drainage; POS, postoperative symptoms; PPCs, postoperative pulmonary complications.

The incidence of total postoperative complications was 17.6% (27/153) in the preoperative SARS‐CoV‐2 infected patients. When stratified by time from SARS‐CoV‐2 diagnosis to surgery, the incidences were as follows: 27.8% (10/36) 0–2 weeks; 16.7% (9/54) 3–4 weeks; 20.7% (6/29) 5–6 weeks; and 5.9% (2/34) at ≥7 weeks. The incidence of total postoperative complications was 11.4% (10/88) in noninfected patients before surgery (Table 2). Compared with patients without SARS‐CoV‐2 infection before surgery, patients with preoperative SARS‐CoV‐2 infection diagnosed 0–2 weeks before surgery had significantly higher incidence of total postoperative complications (OR, 3.00; 95% CI, 1.12–8.01; *p* = 0.028). However, there was no significant difference in total postoperative complications in patients who had surgery at 3–4 weeks, 5–6 weeks, and more than 7 weeks after SARS‐CoV‐2 diagnosis compared with patients without preoperative SARS‐CoV‐2 infection (Figure 2).

In the adjusted analyses, preoperative SARS‐CoV‐2 infection diagnosed 0–2 weeks before surgery remained an independent risk factor of increased total postoperative complications (OR, 5.10; 95% CI, 1.61–16.12; *p* = 0.006). Besides, smoking (OR, 8.05; 95% CI, 2.29–28.36; *p* = 0.001), and sub‐lobectomy (OR, 2.53; 95% CI, 1.09–5.85; *p* = 0.030) were also associated with increased total postoperative complications (Table 3).

**TABLE 3:** Unadjusted and adjusted model for total postoperative complications in all patients. Values are odds ratio (OR) (95% CI).

	Unadjusted		Adjusted	
	OR (95% CI)	*p*‐value	OR (95% CI)	*p*‐value
Age (years)				
<60	Reference	–		
≥60	1.56 (0.76–3.21)	0.226		
Gender				
Female	Reference	–	Reference	–
Male	2.36 (1.16–4.80)	0.018	0.42 (0.12–1.43)	0.163
CCI				
0	Reference	–		
1	1.94 (0.85–4.42)	0.114		
≥2	3.40 (0.80–14.49)	0.099		
ASA physical status				
1–2	Reference	–		
≥3	2.13 (0.64–7.08)	0.755		
BMI				
18.5–25	Reference	–		
<18.5	0.60 (0.12–3.06)	0.541		
>25	0.69 (0.12–3.85)	0.667		
Smoking history				
Nonsmoker	Reference	–	Reference	–
Current or former smokers	4.33 (2.08–8.99)	0.000	8.05 (2.29–28.36)	0.001
COVID‐19 symptoms				
Asymptomatic	Reference	–		
Symptomatic	2.49 (0.31–20.13)	0.393		
Operation side				
Left side	Reference	–		
Right side	1.89 (0.85–4.21)	0.121		
Surgical modalities				
Lobectomy	Reference	–	Reference	–
Sub‐lobectomy	3.53 (1.70–7.37)	0.001	2.53 (1.09–5.85)	0.030
Histologic types				
Adenocarcinoma	Reference	–		
Non‐adenocarcinoma	1.02 (0.42–2.50)	0.967		
pT stage				
T1	Reference	–	Reference	–
T2‐T3	2.96 (1.23–7.14)	0.016	1.13 (0.29–4.39)	0.858
pTNM status				
Stage I	Reference	–	Reference	–
Stage II–III	4.40 (1.32–14.70)	0.016	5.34 (0.79–36.15)	0.086
Preoperative SARS‐CoV‐2 by timing of preoperative diagnosis				
No diagnosis	Reference	–	Reference	–
≤2 weeks	3.00 (1.12–8.01)	0.028	5.10 (1.61–16.12)	0.006
3–4 weeks	1.56 (0.59–4.13)	0.370	2.38 (0.81–7.01)	0.117
5–6 weeks	2.04 (0.69–6.20)	0.211	2.32 (0.68–7.92)	0.179
≥7 weeks	0.49 (0.10–2.35)	0.371	0.88 (0.17–4.69)	0.883

Abbreviations: ASA, American Society of Anesthesiologists; BMI, body mass index; CCI, Charlson Comorbidity Index; COVID‐19, coronavirus disease 2019; SARS‐CoV‐2, the severe acute respiratory syndrome coronavirus 2.

PPCs is very important observation index of thoracic surgery, we found that compared with patients without preoperative SARS‐CoV‐2 infection (4/88, 4.5%), the incidence of PPCs in patients who had surgery at 0–2 weeks after SARS‐CoV‐2 diagnosis (6/36, 16.7%) were also significant difference (OR, 4.20; 95% CI, 1.11–15.91; *p* = 0.035). For patients having surgery >2 weeks after SARS‐CoV‐2 diagnosis, the incidences of PPCs were similar to those without preoperative SARS‐CoV‐2 infection (Figure 2).

**FIGURE 3:**
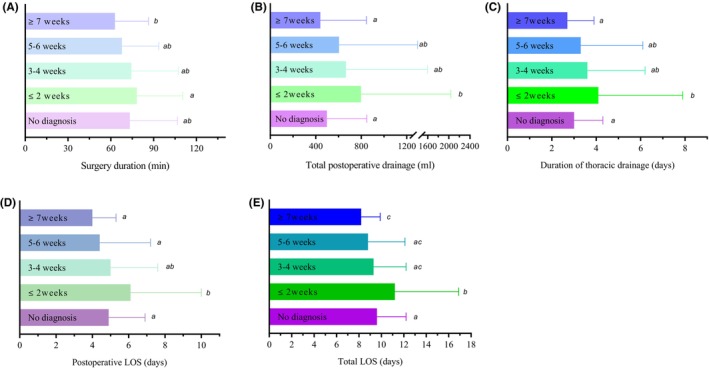
Comparison of perioperative outcomes between patients with and without preoperative SARS‐CoV‐2 infection diagnosed (preoperative infected patients grouped by timing of diagnosis prior to surgery). (A) Surgery duration (min); (B) total postoperative drainage (mL); (C) duration of thoracic drainage (days); (D) total LOS (days); and (E) postoperative LOS (days). LOS, length of hospital stay.

In addition, the total postoperative drainage was significant more in patients with preoperative SARS‐CoV‐2 infection diagnosed 0–2 weeks before surgery than that in noninfected patients before surgery (796.81 ± 1239.6 mL vs. 497.4 ± 351.1 mL, *p* = 0.045). We also found that patients having surgery 0–2 weeks after SARS‐CoV‐2 diagnosis had significantly longer in total LOS (11.2 ± 5.7 vs. 9.6 ± 2.6 days, *p* = 0.016), postoperative LOS (6.1 ± 3.9 vs. 4.9 ± 2.0 days, *p* = 0.023) and the duration of thoracic drainage (4.1 ± 3.8 vs. 3.0 ± 1.3 days, *p* = 0.015) compared with patients who did not have SARS‐CoV‐2 infection before surgery. However, there was no significant difference in the above outcomes between patients operated >2 weeks after SARS‐CoV‐2 infection and patients without SARS‐CoV‐2 infection before surgery (Table 2 and Figure 3).

Surgery duration, 30‐day and 90‐day postoperative symptoms were important observation index for lung surgery patients. There was no significant difference in these indexes between patients with preoperative SARS‐CoV‐2 infection diagnosed within 2 weeks before surgery and patients who did not have SARS‐CoV‐2 infection before surgery (Figures 2 and 3). However, there were significantly higher incidence of 30‐day postoperative symptoms and longer surgery duration in patients who had surgery within 2 weeks after SARS‐CoV‐2 diagnosis compared with patients diagnosed with SARS‐CoV‐2 infection more than 7 weeks before surgery (Figures 2 and 3).

## 4 DISCUSSION

The SARS‐CoV‐2 pandemic has severely affected the treatment of lung cancer patients, especially surgical procedures.^25^ Individuals diagnosed with lung cancer are at an increased risk of SARS‐CoV‐2 infection, resulting in elevated rates of morbidity and mortality compared to the general population.^1^, ^9^ Nonetheless, the heightened risks linked to SARS‐CoV‐2 infection should be weighed against the potential consequences of postponing surgery in individuals based on the clinical stage of their lung cancer.^10^, ^11^, ^12^, ^13^, ^26^ Therefore, thoracic surgeons should recalibrate their approach to ensure that patients receive timely and effective treatment. This study found that NSCLC patients who underwent lung resection surgery within 2 weeks of SARS‐CoV‐2 diagnosis were at an increased risk of postoperative complications, especially PPCs. It has direct implications for clinical practice in lung cancer surgery during the pandemic. This study also identified patients with smoking history or receiving sub‐lobectomy as being more vulnerable to adverse outcomes. As far as we know, this is the first study to provide evidence regarding the optimal timing of lung resection surgery and assessing early outcomes after surgery in NSCLC patients with SARS‐CoV‐2 infection.

In the study, the incidence of total postoperative complications, especially PPCs, were significantly higher in patients who had surgery within 2 weeks after SARS‐CoV‐2 diagnosis than in patients without infection. Our study findings show that preoperative SARS‐CoV‐2 infection increases the risk of PPCs is in line with previous works.^1^, ^2^, ^3^, ^4^ As the most important postoperative complications, PPCs have been shown to be associated with a considerable increase in mortality and LOS.^23^, ^27^ Correspondingly, both the total LOS and postoperative LOS were significantly longer in patients having surgery within 2 weeks after SARS‐CoV‐2 diagnosis than in noninfected patients before surgery in this study. However, there was no significant difference in total postoperative complications, PPCs, LOS and the duration of thoracic drainage between patients with preoperative SARS‐CoV‐2 infection diagnosed more than 2 weeks before surgery and noninfected patients. Additionally, previous studies have shown that postoperative outcomes in SARS‐CoV‐2‐infected patients either receive emergency or major surgery are substantially worse than pre‐pandemic baseline rates of mortality and pulmonary complications^1^, ^2^; these studies identified men, patients aged 70 years or older, those with comorbidities (ASA Grades 3–5), those having cancer surgery, and those in need of emergency or major elective surgery are at particularly high risk of mortality.^1^ However, the total postoperative complications and PPCs increased significantly among patients who had preoperative SARS‐CoV‐2 infection diagnosed within 14 days before surgery, but not after, although our participants were all NSCLC patients, there were no deaths in this study, this is slightly different from previous studies,^2^ that probably because a small sample size in this study, all patients in the study had negative preoperative SARS‐CoV‐2 nucleic acid tests and had no symptoms of COVID‐19 or had fully recovered without any related symptoms after the infection before surgery, that a large proportion of the patients were under 60 years of age and ASA physical status 1–2, and all of them underwent minimally invasive thoracoscopic surgery. In addition, the patients admitted to the hospital was in compliance with national and local epidemic management policies as well as clinical guidelines.

In this study, some patients still had symptoms at 30 days after surgery, but the incidence decreased in patients who had surgery at 5–6 weeks after SARS‐CoV‐2 diagnosis before surgery, and significantly decreased in patients with preoperative SARS‐CoV‐2 infection diagnosed more than 7 weeks before surgery. This might be explained by the following reasons, 30‐day postoperative symptoms are not only lung surgery‐related, but consists of lung surgery‐related and COVID‐19 related,^24^ also 18 patients who were not diagnosed with SARS‐CoV‐2 infection before surgery but had SARS‐CoV‐2 infection within postoperative 30 days were not excluded in our study, and thus the incidence of 30‐day postoperative symptoms was high in patients without preoperative SARS‐CoV‐2 infection. However, there was no difference in 90‐day postoperative symptoms, which occurred in only a small number of patients, among the groups. The incidence of 90‐day postoperative symptoms was similar with 30‐day postoperative symptoms in patients with preoperative SARS‐CoV‐2 infection diagnosed ≥7 weeks before surgery, which were consistent with long COVID.^28^, ^29^ Moreover, SARS‐CoV‐2 infection does not appear to complicate surgical procedures in NSCLC patients, since there was no significant increase in operation duration in patients diagnosed with preoperative SARS‐CoV‐2 infection compared with no diagnosed before surgery.

Besides timing of surgery, our study revealed some other factors related to the incidence of postoperative complications. We found that smoking was an important risk factor for postoperative complications, especially PPCs, for surgical NSCLC patients. Previous studies also suggested that current smokers who underwent lung surgery had an increased risk of death compared with never‐smokers,^30^, ^31^ smoking may increase the risk of respiratory tract infections by impairing the immune system via affecting the macrophage and cytokine response, and hence the ability to contain infection.^31^ In addition, the higher incidence of total postoperative complications after sub‐lobectomy in this study result from prolonged chest drainage or air leakage, which is consistent with previous study.^32^

This study has some limitations. First of all, it is a single center study in China and the sample size is relatively small. Second, false negative results of PCR tests may occur for sampling error or timing of sample, also because initially negative tests becoming positive with subsequent testing. Third, data were collected in hospitals with ongoing SARS‐CoV‐2 infection outbreaks in December, 2022, and most cases were at an early stage of NSCLC in this study. Selection bias may be present in this study because we abided by national and local epidemic management policies as well as clinical guidelines. Given above reasons, our findings still need to be confirmed by further international multicenter studies with large sample size.

## 5 CONCLUSION

This retrospective cohort study provided robust data regarding the early postoperative outcomes of NSCLC surgery following SARS‐CoV‐2 infection, and documents that the incidence of total postoperative complications, especially PPCs, increase in patients with preoperative SARS‐CoV‐2 infection diagnosed within 2 weeks before surgery. However, the SARS‐CoV‐2 infection did not complicate surgical procedures for lung cancer. Our findings suggest that lung resection surgery should be postponed at least 2 weeks after SARS‐CoV‐2 infection for NSCLC patients.

## AUTHOR CONTRIBUTIONS

**Yanbo Yang:** Data curation (equal); formal analysis (lead); investigation (lead); methodology (lead); writing – original draft (lead); writing – review and editing (lead). **Lingli Niu:** Project administration (supporting); resources (lead); supervision (lead). **Yunke Zhu:** Supervision (supporting); validation (lead); writing – review and editing (supporting). **Zhu Wu:** Supervision (equal); validation (equal); writing – review and editing (supporting). **Liang Xia:** Formal analysis (equal); funding acquisition (lead); writing – review and editing (supporting). **Congjia Xiao:** Investigation (supporting); resources (equal). **Xu Shen:** Investigation (equal); resources (supporting). **Xin Xiao:** Formal analysis (supporting). **Conglin Tian:** Investigation (supporting); resources (supporting). **feng lin:** Conceptualization (lead); methodology (supporting); project administration (lead); writing – review and editing (equal).

## FUNDING INFORMATION

This study was supported by the Science and Technology Project of the Health Commission of Sichuan Province, China (No. 21PJ008 to Liang Xia) and Key Projects of Sichuan Province (No. 2022YFS0208 to Liang Xia).

## CONFLICT OF INTEREST STATEMENT

All authors have no conflicts of interest or financial ties to disclose.

## ETHICS STATEMENT

This study was compliant with guidelines for the reporting of observational studies, and approved by the Institutional Review Board (IRB) of West China Hospital, Sichuan University.

## THE APPROVAL NUMBER

2023‐1626.

## Data Availability

The data sets generated during and/or analyzed during the current study are available from the corresponding author on reasonable request.
